# Effect of Gamma Irradiation on Structural and Biological Properties of a PLGA-PEG-Hydroxyapatite Composite

**DOI:** 10.1155/2014/420616

**Published:** 2014-09-08

**Authors:** Sima Shahabi, Farhood Najafi, Abbas Majdabadi, Tabassom Hooshmand, Masoumeh Haghbin Nazarpak, Batool Karimi, Seyyed Mostafa Fatemi

**Affiliations:** ^1^Department of Dental Biomaterials, School of Dentistry, Tehran University of Medical Sciences, End of North Kargar Street, Tehran, Iran; ^2^Department of Resin and Additives, Institute for Color Science and Technology, 55 Vafamanesh Street, HosseinabadSquare, P.O. Box 16765-654, Tehran, Iran; ^3^Laser Research Center of Dentistry, School of Dentistry, Tehran University of Medical Sciences, End of North Kargar Street, Tehran, Iran; ^4^New Technologies Research Center (NTRC), Amirkabir University of Technology, 424 Hafez Avenue, Tehran, Iran; ^5^Iranian Center for Medical Laser Research, ACECR Tehran Medical Sciences Branch, Tehran, Iran

## Abstract

Gamma irradiation is able to affect various structural and biological properties of biomaterials In this study, a composite of Hap/PLGA-PEG and their ingredients were submitted to gamma irradiation doses of 25 and 50 KGy. Various properties such as molecular weight (GPC), thermal behavior (DSC), wettability (contact angle), cell viability (MTT assay), and alkaline phosphatase activity were studied for the composites and each of their ingredients. The results showed a decrease in molecular weight of copolymer with no change in the glass transition and melting temperatures after gamma irradiation. In general gamma irradiation can increase the activation energy Δ*H* of the composites and their ingredients. While gamma irradiation had no effect on the wettability of copolymer samples, there was a significant decrease in contact angle of hydroxyapatite and composites with increase in gamma irradiation dose. This study showed an increase in biocompatibility of hydroxyapatite with gamma irradiation with no significant effect on cell viability in copolymer and composite samples. In spite of the fact that no change occurred in alkaline phosphatase activity of composite samples, results indicated a decrease in alkaline phosphatase activity in irradiated hydroxyapatites. These effects on the properties of PLGA-PEG-hydroxyapatite can enhance the composite application as a biomaterial.

## 1. Introduction

Biodegradable polymers and bioactive ceramics are widely used for bone tissue engineering. They combine to form a variety of composites which are able to achieve the strength of inorganic phase with the formability of organic phase at the same time [1]. For better biocompatibility and applicability, the structure of these biomaterials can be affected by different physical, chemical, and biological modification which can alter different aspects of biomaterials such as degradation, hydrophilicity, bioactivity, and sterility [2, 3]. Gamma irradiation has been frequently used for sterilization of biomedical materials [4]. Structural and biological properties of materials can be affected by gamma irradiation as well [5, 6]. In a dose dependent manner, gamma irradiation is able to reduce molecular weights of polymers due to radical production [7]. On the other hand higher doses of gamma irradiation can even induce crosslinking in polymers [8]. Some structural changes in bioceramics such as hydroxyapatite may occur after gamma irradiation like an increase in their enthalpy [9]. Biocompatibility and bioactivity of biomaterials can be affected, and it is shown that the bioconductivity and absorption of these materials are dependent upon irradiation dose [10].

Biodegradable polymers such as poly(ethylene glycol) and poly(D,L lactic and glycolic acid) and their various copolymers are commonly used in tissue engineering, drug delivery, and biological researches [1] and effect of gamma irradiation on them has been studied in many researches [4, 11, 12]. However there are few studies which investigated various properties simultaneously and some properties such as enzyme activity are rarely studied.

The aim of this study is to investigate the impact of different doses of gamma irradiation on structural and biological properties of a PLGA-PEG-hydroxyapatite composite and each of its ingredients. As 25 KGy is the most common dose of gamma irradiation for serialization of biomedical products [13], in this study, after preparation of hydroxyapatite, copolymer, and their composite, they were submitted to 25 and 50 KGy gamma irradiation, and various properties including changes in molecular weight, thermal behavior, wettability, cell viability, and alkaline phosphatase activity of the composite and each of its ingredients were studied separately.

## 2. Materials and Methods

### 2.1. Materials

#### 2.1.1. Hydroxyapatite

**Figure 1:**
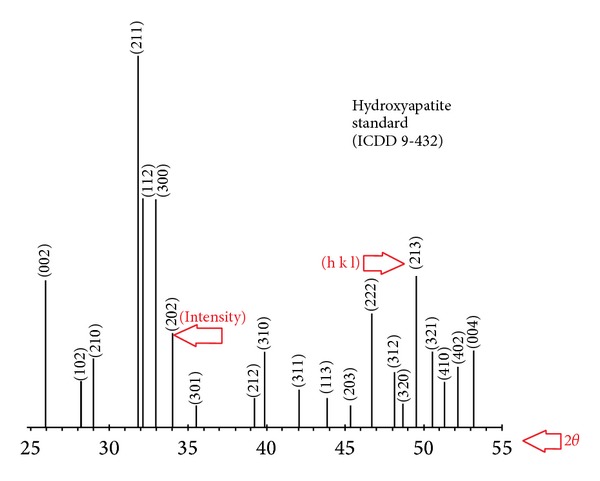
The standard XRD peaks of hydroxyapatite (based on ICDD 9–432).

**Figure 2:**
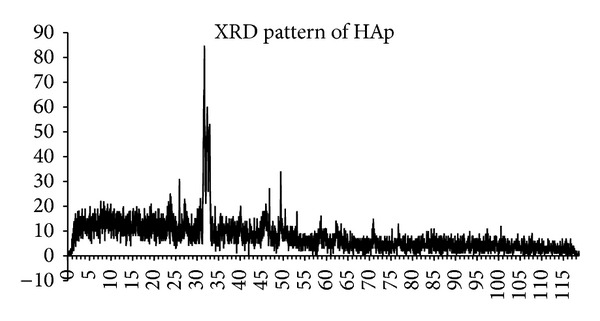
The XRD pattern of synthesized HAp.

A common wet chemical method was applied in hydroxyapatite preparation in which calcium source was calcium nitrate tetrahydrate [Ca(NO_3_)_2_ + 4H_2_O] (C1396 SIGMA-ALDRICH) and phosphate source was diammonium hydrogen phosphate [(NH_4_)_2_HPO_4_] (A5764 SIGMA-ALDRICH) with Ca/P ratio of 1.67. The pH during the reaction was kept at 10.5–11 using NaOH (306576 SIGMA-ALDRICH) 1 N solution. The precipitates were dried and calcinated in 800°C for 2 hours. The formation of hydroxyapatite was characterized by XRD analysis. The standard XRD peaks of hydroxyapatite (based on ICDD 9–432) are shown in Figure 1 and the XRD pattern of synthesized HAp is shown in Figure 2.  Table 1 shows the main peaks and their intensity of standard and synthesized sample.

**Figure 3:**
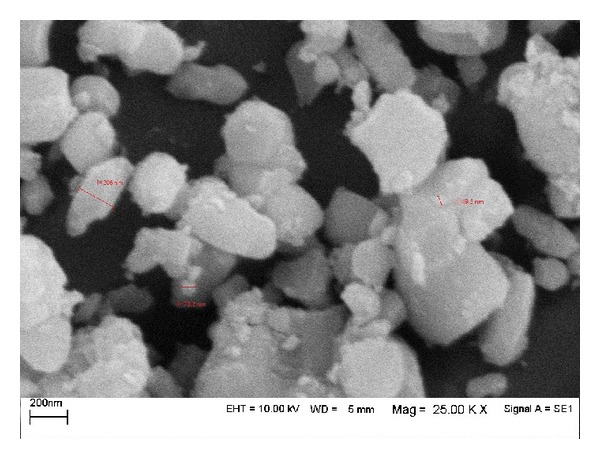
SEM of synthesized HAp and particle measurement.

The mean particle size of HAp in this method was 50 to 200 nm as measured in Figure 3. Also according to the XRD data of the synthesised HAp, the minimum size of the hydroxyapatite crystallites was also calculated using Scherrer equation (19.18 nm).

#### 2.1.2. PLGA-PEG-PLGA Copolymer

A synthesized copolymer of poly(lactic/glycolic) (80 : 20) (W261106 and 124737 SIGMA-ALDRICH) and polyethylene glycol (*M*_*w*_: 2000) (84797 SIGMA) in which the molar weight ratio of PLGA/PEG was 95 : 5 was used in this study. NMR and FTIR characterization of chemical structure of the copolymer was done (not included). The copolymer was poured in small stainless steel split molds (8 mm^3^ cubes) for further treatments and tests.

#### 2.1.3. Composites

**Figure 4:**
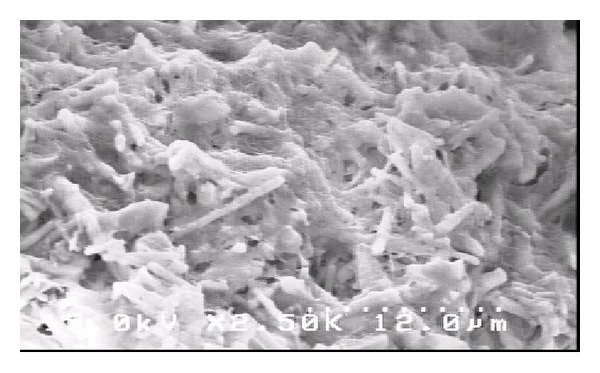
SEM micrograph of the matrix of copolymer with dispersed phase of hydroxyapatite needle-shaped particles.

The weight ratio of HAp/copolymer in the composite was 25 : 75, and the composites samples were prepared by dissolving and stirring proper amount of milled and sieved hydroxyapatite and copolymer in tetrahydrofuran (4 hours) to make a homogenous dispersion. The mixture was poured in the same small molds (8 mm^3^ cubes). The solvent was eliminated by vacuum dry (−0.5 bar) for 12 hours. As shown in Figure 4, there was a matrix of copolymer with dispersed phase of hydroxyapatite needle-shaped particles.

### 2.2. Gamma Irradiation

The hydroxyapatite, copolymer, and composite samples were randomly assigned to three groups with irradiation doses of 0, 25, and 50 kGy of gamma irradiation.

Using ^60^Co as irradiation source, the irradiation was made at room temperature, in presence of air. With irradiation dose rate of 1kGy per hour, the temperature of sample was kept unchanged (monitored by a thermometer) during irradiation to prevent structural changes.

### 2.3. Methods of Assessment

#### 2.3.1. Gel Permeation Chromatography

The molecular weights of copolymers before and after irradiation were determined by gel permeation chromatography (GPC). The GPC instrument brand was a Shimadzu 6-A, consisting of Waters Ultrastyragel 10^4^, 10^3^, and 500 Å columns and refractive index detector. The standard material in this system was polystyrene and tetrahydrofuran (THF) was the mobile phase with flow rate of 1 mL/min at temperature of 40°C.

#### 2.3.2. Differential Scanning Calorimetry

Thermal behaviors of copolymers, hydroxyapatites, and composites were studied before and after irradiation, using a Mettler DSC 823 (Mettler Toledo GmbH, Switzerland) with Mettler Star Software, version 9.01. The samples were heated from 0°C to 110°C with a scan rate of 10°C/min.

#### 2.3.3. Wettability and Contact Angle

**Figure 5:**
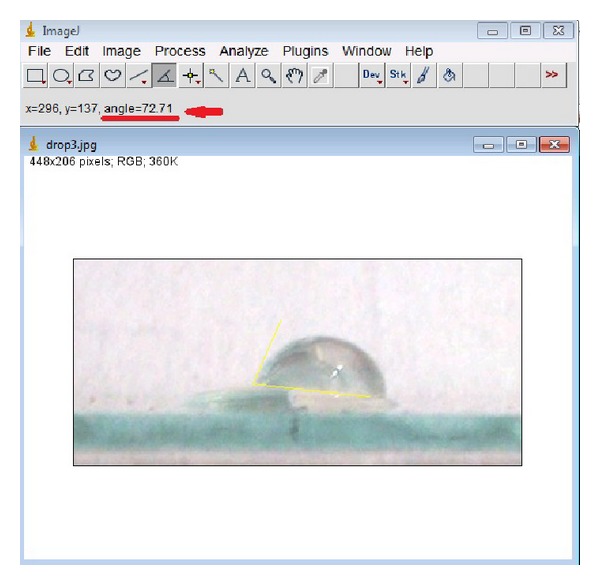
Direct measurement of contact angle using Imagej 1.64r software.

**Figure 6:**
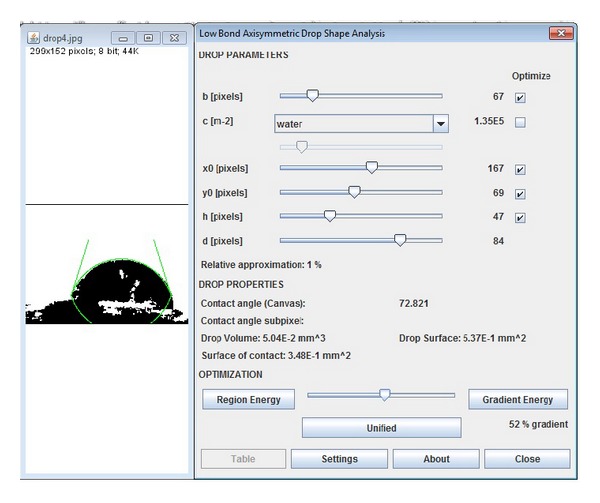
Measurement of contact angle using drop analysis plug-in of Imagej 1.64r software of the same sample.

By measuring the contact angle of distilled water (after 10 min of being in an ultrasonic bath in ethanol), the samples were analyzed for wettability test before and after irradiation. Contact angles were assessed directly by measuring the angle formed between the solid and the tangent to the drop surface using drop analysis plug-in of Imagej 1.64r software. Figures 5 and 6 show direct measurement and drop analysis plug-in measurement of the software for the same sample, respectively.

#### 2.3.4. Cell Viability (MTT Assay)

MTT assay was performed for evaluation of cells' viability and samples' biocompatibility using L929 fibroblasts (obtained from National Cell Bank of Iran (NCBI)) in days 3, 7, and 14 of culture. In this analysis, 5 mg of each sample material was placed in a well (in a 96-multiwell plate) in contact with cells (20000 cells in each well). At the time of evaluation (days 3, 7, and 14), the medium on cells was discarded, washed twice with PBS, and replaced by MTT-containing medium. The plate was incubated at 37°C for 4 h. Then the MTT solution was discarded and without washing, DMSO was added to solve the formazan formed. The sample was left for 15 min. The cells plate was transferred to the Eliza reader and the absorbance at 570 nm was measured. The results were expressed as percent of viable cells in comparison with the group of fibroblasts which were in contact with no materials serving as control group.

#### 2.3.5. Bioactivity (Alkaline Phosphatase Activity)

The samples were analyzed for alkaline phosphatase enzyme activity using Mg-63 human cells (obtained from National Cell Bank of Iran (NCBI)) in days 7 and 14 of contact. In sum, 5 mg of each sample material was placed in a well (in a 96-multiwell plate) in contact with cells (20000 cells in each well). At the time of evaluation (days 3, 7, and 14), the medium on cells was discarded and the cells were washed with PBS twice before addition of lysis buffer (1.5 M Tris-HCl (pH 9), 0.5 M MgCl_2_–6H_2_O, and 0.2% Triton X-100). The cells then were sonicated for 30 seconds. Then the substrate solution of 4-nitrophenyl phosphate (PNPP) buffered with diethanolamine was added to cell wells and incubated for 20 minutes at 37°C. Subsequently, 1 N sodium hydroxide was added to the samples to stop the reaction. Absorbance of the resultant P-nitrophenol was measured by the Eliza reader at 405 nm to express alkaline phosphatase activity. The results were expressed as percent of enzyme activity in comparison with the group of osteoblasts which were in contact with no materials serving as control group.

### 2.4. Statistical Analysis

Statistical analysis of data was performed using PASW Statistics 18 for analysis of variance. The *P* value was considered to be less than 0.05.

## 3. Results and Discussion

### 3.1. Gel Permeation Chromatography

The results of GPC are shown in Table 1. As expected, chain scissoring and a decrease in molecular weight are the main impact of gamma irradiation on copolymers in a dose dependent manner.

Scission yield *G*(*S*) and crosslinking yield *G*(*X*) have also been calculated from the following combination of equations and are shown in Table 2 [14]:

(1) $$\begin{array}{c} \frac{1}{M_{nD}}=\frac{1}{M_{n0}}+1.04\times 10^{-10}\left[G\left(S\right)-G\left(X\right)\right]D,\\ \frac{1}{M_{wD}}=\frac{1}{M_{w0}}+5.18\times 10^{-11}\left[G\left(S\right)-4G\left(X\right)\right]D. \end{array}$$

In the above equations *D* stands for s irradiation dose in Gy (0 for nonirradiated samples). *M*_*n*0_ and *M*_*w*0_ stand for the weight and number average molecular weights of nonirradiated samples. *M*_*wD*_ and *M*_*nD*_ are the corresponding values after irradiation.

Scission yield *G*(*S*) and crosslinking yield *G*(*X*) also show that the chain scission was the dominant phenomenon in the copolymers after irradiation. Moreover, the predominant event was random chain scission with respect to unzipping.

Our results showed that there was a significant decrease in molecular weight of polymer (both *M*_*w*_ and *M*_*n*_) with gamma irradiation which was in agreement with the results of the previous studies [15–19]. Since the ratio of *G*(*S*)/*G*(*X*) is greater than 4, the chain scission was the dominant mechanism in our samples, though there might have been some crosslinking in polymer chains [20]. Our results showed no significant change in polydispersity index of copolymers after gamma irradiation with these doses of gamma irradiation, which disagreed with those studies that had suggested that *M*_*n*_ decreases more than *M*_*w*_ with polymer exposure to gamma irradiation and the polydispersity index may increase [21, 22].

### 3.2. Differential Scanning Calorimetry

**Figure 7:**
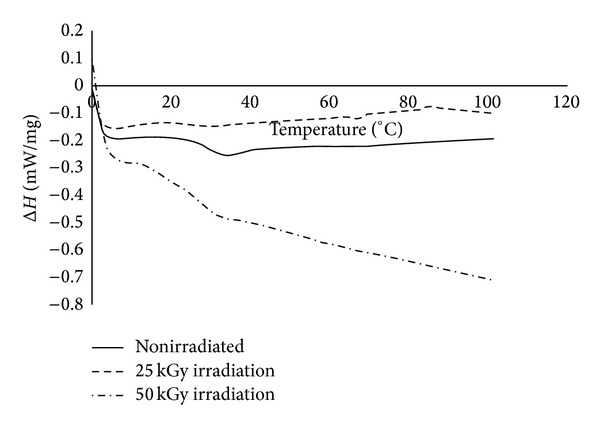
DSC diagram of copolymers irradiated with 0, 25, and 50 KGy doses of gamma irradiation.

As shown in Figure 7 and indexed in Table 3, gamma irradiation resulted in an insignificant decrease in the glass transition and melting temperatures of copolymers with the increase in dose, which is consistent with other studies [23–26].

Likewise an insignificant difference was shown between total activation energy Δ*H* for glass transition and melting of 0 and 25 KGy gamma irradiated copolymers; meanwhile there was a significant increase for 50 KGy gamma irradiated copolymers.

Our results showed that the activation energy Δ*H* of nonirradiated and 25 KGy and 50 KGy gamma irradiated copolymer samples from 0°C to 110°C was −23.33, −13.32, and −59.43 mw/mg, respectively. This is in agreement with existing data in the literature that, regardless of a major decrease in the molecular weights of polymers, even a decrease in Δ*H* at low doses may be observed while at higher doses an increase in activation energy occurs probably due to energy relaxation of polymers [26, 27].

**Figure 8:**
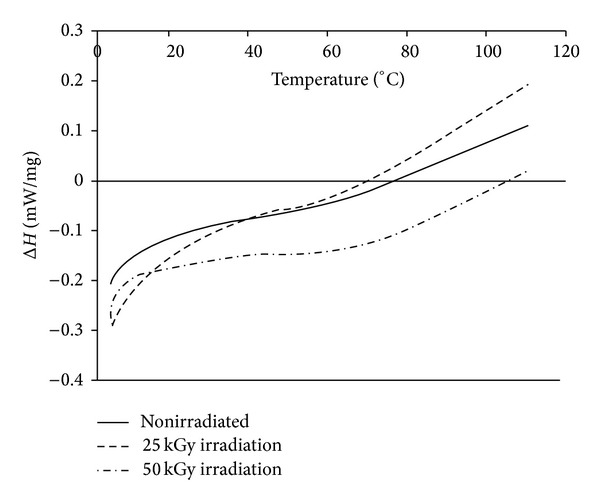
DSC diagram of hydroxyapatite irradiated with 0, 25, and 50 KGy dose of gamma irradiation.

Figure 8 shows the DSC diagram of hydroxyapatite under different irradiation doses. Although the hydroxyapatite thermal behavior was almost the same in 0 and 25 kGy dose of gamma irradiation, there was a significant increase in activation energy Δ*H* of 50 kGy gamma irradiated group between 0 and 110°C.

The point of turning the curve from endothermic to exothermic behavior was about 75°C for 0 and 25 kGy gamma irradiated hydroxyapatite samples, and this point increased to 106°C for 50 kGy gamma irradiated group. The total heat in endothermic phase of 0 and 25 kGy gamma irradiated groups was −6.40 and −7.60 mw/mg, respectively, while it was −13.86 mw/mg for 50 kGy gamma irradiated group. The activation energy Δ*H* of nonirradiated and 25 KGy and 50 KGy gamma irradiated hydroxyapatite samples from 0°C to 110°C was −4.51, −3.62, and −13.82 mw/mg, respectively. The data in the literature which is in agreement with this result shows that the gamma irradiation of bone as a composite system of collagen, hydroxyapatite, and water can increase its enthalpy [9].

**Figure 9:**
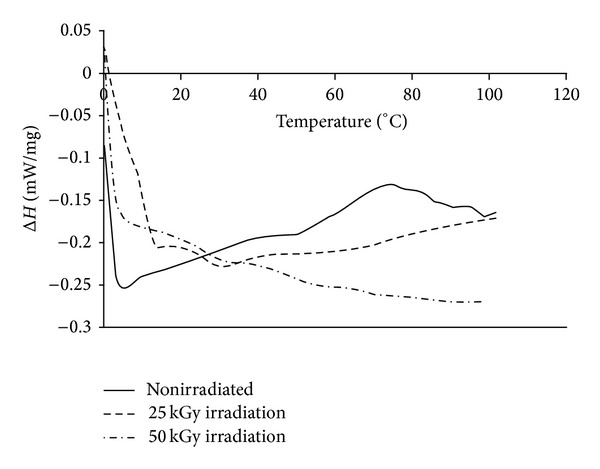
DSC diagram of composites irradiated with 0, 25, and 50 KGy dose of gamma irradiation.

Figure 9 shows the DSC diagram of the composites under different irradiation doses. Owing to the HAp/polymer ratio of 25 : 75, it can be seen that the thermal behavior of composite is also similar to its ingredients. There was an insignificant difference between nonirradiated and 25 KGy gamma irradiated composite samples, while the 50 KGy irradiated sample showed a significant endothermic behavior. The activation energy Δ*H* of nonirradiated and 25 KGy and 50 KGy gamma irradiated composite samples from 0°C to 110°C was −20.76, −18.85, and −26.3 mw/mg, respectively.

Our study also showed an insignificant decrease in activation energy Δ*H* of 25 KGy gamma irradiated copolymer and composite samples from 0°C to 110°C and an increase in activation energy Δ*H* of 50 KGy gamma irradiated samples while one study mentioned that the activation energy of irradiated composite of polymethyl methacrylate (PMMA) and hydroxyapatite was higher than irradiated unfilled polymer due to stabilizing effect of hydroxyapatite [28, 29].

### 3.3. Wettability and Contact Angle

**Figure 10:**
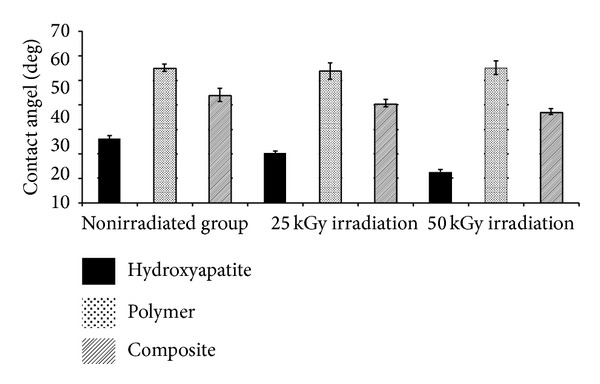
Contact angle of hydroxyapatite, copolymers, and composites irradiated with 0, 25, and 50 KGy doses of gamma irradiation.

The results of contact angle measurement of hydroxyapatite, copolymer, and composite under various irradiation doses are shown in Figure 10 and Table 4.

There was a significant decrease in contact angle of hydroxyapatite in a dose dependent manner. Our results are not in line with the finding of a study performed by Colaço et al. in which there was no change in contact angles after gamma irradiation on plasma sprayed hydroxyapatite [30].

There was no significant difference between contact angles of copolymers and our results differ from those of a recent study in which there was a significant change in contact angles after radiation probably due to different doses of gamma irradiation [31].

The composites have shown a little but statistically significant decrease in contact angle with increase in gamma irradiation dose owing to the presence of hydroxyapatite which enhances the wettability.

### 3.4. Cell Viability (MTT Assay)

There were some significant changes in percent of viable cells and biocompatibility of materials under various doses of irradiation in days 3, 7, and 14. The whole results are shown in Table 5.

In copolymers and composites there was no significant difference between cell viability of irradiated and nonirradiated groups. The biocompatibility of copolymers decreases through time, due to their acidic degradation byproducts.

Due to buffering effect of hydroxyapatite, the cell viability of composite samples was significantly more than copolymer groups. Our results are not consistent with some other studies in which less biocompatibility was observed in the irradiated polymers [32], although we were in agreement with a recent study in which there was no significant change in cell viability in gamma irradiated composites [33].

In general gamma irradiated hydroxyapatite groups showed significantly more viable cell percentage in comparison with nonirradiated group and the biocompatibility and cell viability did not differ significantly in a time-dependent manner.

Our results confirm the data already available in the literature as high energy ion beam irradiation has been shown to enhance the biocompatibility and bioactivity of hydroxyapatite with a decrease in crystallite size on irradiation [25, 34, 35]. Low energy ion beams have also improved wettability, bioactivity, and protein absorption without major structural changes [36, 37]. An increase in crystallinity of polylactic acid/hydroxyapatite nanocomposite films has also been shown after gamma irradiation [38, 39].

In hydroxyapatite it can be related to the structural changes after irradiation such as a decrease in crystallite size, which may enhance cell viability as some other studies have shown better cell viability on the smaller-sized crystals of HAp [40, 41].

### 3.5. Bioactivity (Alkaline Phosphatase Activity)

Changes in the bioactivity (alkaline phosphatase activity) of the materials under various doses of irradiation in days 7 and 14 are shown in Table 6.

Irradiated copolymers showed a significant increase of alkaline phosphatase activity in comparison with nonirradiated group in the first week, but the enzyme activity decreased through time in all groups and there was no significant difference between test groups in day 14. All copolymer samples reduced the enzyme activity in comparison with control group (cells without material).

The irradiated hydroxyapatite groups resulted in less alkaline phosphatase activity in comparison with nonirradiated groups and there was no significant difference between 25 and 50 KGy gamma irradiated samples. In all samples the enzyme activity was significantly enhanced compared to the control group (cells without material).

The bioactivity of all samples increased significantly in 14th day in comparison with 7th day.

In composite groups there was no difference between bioactivity of nonirradiated and irradiated groups in the first week, and the enzyme activity decreased through time in all groups. All composite samples reduced the enzyme activity in comparison with control group (cells without material) which was insignificant. Due to presence of hydroxyapatite, composites groups have shown higher alkaline phosphatase activity than copolymer groups but lower than the control group.

The effect of gamma irradiation on alkaline phosphatase activity of cells in contact with irradiated materials has rarely been studied. Our results show a decrease in enzyme activity in irradiated hydroxyapatite and an increase in alkaline phosphatase activity in irradiated copolymer samples with no change in composite group. Like other studies ALP activity increased in a time-dependent manner due to osteoconductive effect of these composites [42].

If we want to consider the results of cell viability (MTT assay) along with alkaline phosphatase activity, it should be mentioned that ALP activity indicates mineralization activity of cells and their differentiation toward osteoblastic phenotype [43]. Meanwhile MTT assay presents general metabolism of cells and their viability. So, in some situations, the enzyme activity may be enhanced along with reduction in cell metabolism. Even biocompatible biomaterials like HAp, PLGA, and PEG are able to decrease cell metabolism in comparison with control group, as seen in our study and many others, but meanwhile some of osteoconductive biomaterials like calcium phosphate bioceramics have an improving effect on alkaline phosphatase activity. The effect of gamma irradiation on copolymers and composites can be expressed as no change in biocompatibility and bioactivity. On the other hand HAp becomes more biocompatible and less bioactive by gamma irradiation.

## 4. Conclusion

Gamma irradiation can significantly affect the structural and biological properties of PLGA-PEG-hydroxyapatite composite and its ingredients.

The molecular weight of copolymers (both *M*_*w*_ and *M*_*n*_) decreased in a dose dependent manner. High doses of gamma irradiation resulted in an increase in activation energy of HAp, copolymers, and their composites while lower doses could not affect thermal behavior of the samples significantly. Although gamma rays had no effect on contact angle and wettability of copolymers, they could enhance hydrophilicity of the HAp and composites. And at last, while cell viability and alkaline phosphatase activity of copolymers and composites were not affected by gamma rays, HAp has shown more biocompatible and less bioactive behavior under gamma irradiation.

These changes in the properties of these biomaterials may enhance their application in tissue engineering.

## Figures and Tables

**Table 1:** The main peaks and their intensity of standard (ICDD 9-432) and synthesized HAp sample along with the Miller indices.

*h*	*K*	*l*	2*θ* ICDD HAp	Peak intensity %	2*θ* synthesized HAp	Peak intensity %
0	0	2	25.879	40	25.893	36.90
2	1	1	31.773	100	31.698	100
1	1	2	32.196	60	32.351	69.43
3	0	0	32.902	60	32.914	63.10
2	0	2	34.048	25	34.169	23.81
3	1	0	39.818	20	39.978	21.43
2	2	2	46.711	30	46.783	32.14
2	1	3	49.468	40	49.409	40.48
3	2	1	50.493	20	50.448	22.62
0	0	4	53.143	20	53.173	21.43

**Table 2:** Weight and number average molecular weights, polydispersity index, scission yield *G*(*S*), and crosslinking yield *G*(*X*) of irradiated and nonirradiated copolymer samples.

Copolymer property	Nonirradiated copolymer	25 KGy irradiated copolymer	50 KGy irradiated copolymer
*M*_*w*_	63.137 KDa	18.551 KDa	16.491 KDa
*M*_*n*_	19.579	6.405	5.674
PDI (*M*_*w*_/*M*_*n*_)	3.224	2.896	2.906
*G*(*S*)	—	35.6	26.84
*G*(*X*)	—	3.2	2.42

**Table 3:** Glass transition and melting temperature of irradiated and nonirradiated copolymer samples along with total energy for them.

Copolymer property	Nonirradiated copolymer	25 KGy irradiated copolymer	50 KGy irradiated copolymer
*T*_*g*_ (glass transition temperature)	27.3°C	26.7°C	25.9°C
*T*_*m*_ (melting temperature)	39.6°C	38°C	37.3°C
Δ*H*_*g*_ (heat of glass transition)	−4.83 mw/mg	−3.24 mw/mg	−6.27 mw/mg
Δ*H*_*m*_ (heat of melting)	−7.4 mw/mg	−5.15 mw/mg	−11.41 mw/mg

**Table 4:** Contact angle of hydroxyapatite, copolymers, and composites irradiated with 0, 25, and 50 KGy doses of gamma irradiation.

Material	Nonirradiated samples	25 KGy irradiated samples	50 KGy irradiated samples
Hydroxyapatite	26.16° ± 2.51°	20.35° ± 1.35°	12.62° ± 1.4°
Copolymer	55.16° ± 2.53°	53.96° ± 5.85°	55.25° ± 2.73°
Composite	44.25° ± 4.53°	42.24° ± 2.58°	37.23° ± 1.96°

**Table 5:** Detailed results of MTT assay (percent of vital cells in comparison to control group) for hydroxyapatite, copolymer, and composite under 0, 25, and 50 KGy doses of gamma irradiation in days 3, 7, and 14.

Material	Nonirradiated samples Percent of vital cells in comparison to control group			25 KGy irradiated samples Percent of vital cells in comparison to control group			50 KGy irradiated samples Percent of vital cells in comparison to control group		
	Day 3	Day 7	Day 14	Day 3	Day 7	Day 14	Day 3	Day 7	Day 14
Hydroxyapatite	47.7 ± 4.66	40.79 ± 10.66	39.43 ± 1.23	64.3 ± 12.8	49.42 ± 7.08	71 ± 15.2	47.28 ± 2	60.77 ± 3.55	65.4 ± 12
Copolymer	32.07 ± 2.02	23.5 ± 5.58	14.68 ± 0.52	40.9 ± 2	17.87 ± 1.98	15.07 ± 1.7	34.6 ± 4.57	17.28 ± 2.44	16.88 ± 2.7
Composite	36.65 ± 0.8	21.44 ± 1.07	24.34 ± 6.13	53.26 ± 22.8	20.37 ± 4.02	23.6 ± 4.3	38.26 ± 3.52	24.61 ± 9.3	22.1 ± 6.2

**Table 6:** Detailed results of alkaline phosphatase activity test (percent of enzyme activity in comparison with control group) for hydroxyapatite, copolymer, and composite under 0, 25, and 50 KGy doses of gamma irradiation in days 3, 7, and 14.

Material	Nonirradiated Percent of enzyme activity in comparison with control group		25 KGy irradiation Percent of enzyme activity in comparison with control group		50 KGy irradiation Percent of enzyme activity in comparison with control group	
	Day 7	Day 14	Day 7	Day 14	Day 7	Day 14
Hydroxyapatite	99.22 ± 4.52	130.2 ± 19.88	87 ± 12.25	105.58 ± 8.54	77.77 ± 3.6	113.72 ± 1.7
Copolymer	60.22 ± 3.65	51.76 ± 2.61	76.88 ± 2.72	64.64 ± 5.67	69.44 ± 3	51.96 ± 6.62
Composite	82.88 ± 1.83	72.84 ± 2.52	81.11 ± 15.29	65.5 ± 1.8	81.88 ± 4.22	67.35 ± 13.7
